# The Evolution of Neuroplasticity and the Effect on Integrated Information

**DOI:** 10.3390/e21050524

**Published:** 2019-05-24

**Authors:** Leigh Sheneman, Jory Schossau, Arend Hintze

**Affiliations:** 1 Department of Computer Science and Engineering, Michigan State University, East Lansing, MI 48823, USA; 2 BEACON-Center for the Study of Evolution in Action, Michigan State University, East Lansing, MI 48823, USA; 3 Department of Integrative Biology Michigan State University, East Lansing, MI 48823, USA

**Keywords:** information integration theory, neuroevolution, autonomous learning

## Abstract

Information integration theory has been developed to quantify consciousness. Since conscious thought requires the integration of information, the degree of this integration can be used as a neural correlate (Φ) with the intent to measure degree of consciousness. Previous research has shown that the ability to integrate information can be improved by Darwinian evolution. The value Φ can change over many generations, and complex tasks require systems with at least a minimum Φ. This work was done using simple animats that were able to remember previous sensory inputs, but were incapable of fundamental change during their lifetime: actions were predetermined or instinctual. Here, we are interested in changes to Φ due to lifetime learning (also known as neuroplasticity). During lifetime learning, the system adapts to perform a task and necessitates a functional change, which in turn could change Φ. One can find arguments to expect one of three possible outcomes: Φ might remain constant, increase, or decrease due to learning. To resolve this, we need to observe systems that learn, but also improve their ability to learn over the many generations that Darwinian evolution requires. Quantifying Φ over the course of evolution, and over the course of their lifetimes, allows us to investigate how the ability to integrate information changes. To measure Φ, the internal states of the system must be experimentally observable. However, these states are notoriously difficult to observe in a natural system. Therefore, we use a computational model that not only evolves virtual agents (animats), but evolves animats to learn during their lifetime. We use this approach to show that a system that improves its performance due to feedback learning increases its ability to integrate information. In addition, we show that a system’s ability to increase Φ correlates with its ability to increase in performance. This suggests that systems that are very plastic regarding Φ learn better than those that are not.

## 1. Introduction

Information Integration Theory [1,2] defines the neural correlate Φ that seeks to quantify how conscious a system is. It does this by measuring the degree to which the components of a system compute more information than the sum of its parts [3]. At the same time, the ability to integrate information is probably beneficial for cognitive systems in general, as it allows these systems to make decisions based on information obtained from multiple environmental sensors and integrate this with knowledge of past outcomes. Imagine a child playing soccer for the first time trying to score a goal. To kick the ball, she must visually locate the ball and determine how to make contact with her foot to move the ball in the desired direction. The cognitive structure she has as a child is drastically different from that of the professional soccer player she might become. We assume that the amount of information integration is different for the child and the professional because the neural structures are different [4], but we are unsure if the amount of information integration increases or decreases with neural change. The child’s cognitive system might need to process a great amount of information while the professional’s cognitive system is already streamlined by training to the point that kicking goals becomes a near unconscious reflex. Alternatively, the professional, being much more accurate and with more experience, might integrate more information than the child.

Information integration has been proposed to be a neural correlate for conscious experience [5] under the assumption that consciousness requires information integration. The neural correlate Φ has undergone many iterations and refinements, with each new iteration also slightly redefining the measurement. Therefore, we will use Φ to refer to the concept of information integration in general, and the special nomenclature when referring to specific versions of Φ, such as: $\Phi^{Max}$, EI, $\Phi_{atomic}$, or $\Phi_{maxH}$ (see below for their definitions). Also, attention is a different process than consciousness [6] and thus Φ should not necessarily correlate with it. Obviously, the task of kicking the ball could also be interpreted in the light of attention. Since we do not have a neural correlate for attention, and our focus is on information integration, we disregard attention for now.

Unfortunately, we do not yet have the ability to thoroughly measure Φ in humans, and thus we must use computational models instead. A lot of previous research [7,8,9,10,11] used animats, which are virtual organisms that are controlled by a Markov Brain [12] (a form of neural networks, MB in short). Since the introduction of information integration theory [4,13,14,15], it has been shown that the amount of information integrated by a system increases over the course of Darwinian evolution [7,8,9]. We have established that task complexity defines a lower bound of Φ [8] for the task to be solved and that more complex environments require more Φ [9]. The use of animats in these investigations has shown that they are technically easy to use, have a small computational footprint, and can be perfectly introspected, making them the ideal surrogate in the absence of natural organisms. However, the interaction between Φ and lifetime learning has not been explored. Here, we evolve animats to perform a learning task, and measure Φ at various stages during their life to answer the question “How does Φ change in a learning organism?”

### 1.1. Markov Brains

MBs are networks of computational logic units (which from now on we will refer to as gates) that are genetically encoded and subject to evolution [7,8,9,11,16,17,18,19,20] (For a detailed description of Markov Brains, see [12]). Conventionally, the MBs contain deterministic and probabilistic gates, each of which can be thought of as a $2^{i}\times 2^{o}$ state transition table, *P*, where *i* is the number of inputs and *o* is the number of outputs. Probabilistic gates are coded so each input is associated with a vector of probabilities determining the likelihood for each possible output to occur. Deterministic logic gates work the same way, except their vector contains a single probability of 1.0 and the rest are 0.0. Each MB is represented by a sequence of numbers that serves as the genome and contains stretches encoding computational units similar to how genes encode proteins. These genes determine the number, type, and the probability tables if necessary. In addition, each gene contains information about how gates are connected to each other and how they interface with sensors or actuators.

Markov Brains built from deterministic and probabilistic logic gates can perform complex computations and can control animats in virtual environments. They can use past experiences to inform future decisions, and form representations about their environment [16]. However, they do not change their composition, connections, or logic, and instead remain static throughout their lives. Changes only occur as a result of mutation, which happens between generations. If we want to study learning and how learning changes ability to integrate information, then the animats need past experiences to change the computation over time. Similar to long-term potentiation (LTP), which strengthens some neural connections and weakens others, here we allow probabilities of logic gates to change based on internal feedback. We pioneered this method by adding so-called *feedback gates* to the repertoire of gates from which Markov Brains can be built. For a detailed description, see Sheneman and Hintze 2017 [21].

In essence, feedback gates have two additional inputs for positive or negative reinforcement. At each update, a feedback gate records which output was mapped to the current input. For positive feedback, the probability that leads to this mapping becomes increased; in the case of negative feedback, this probability becomes decreased. After that, the probability table is normalized so that each row of the probability table sums to 1.0. The strength of the feedback as well as the number of time points the feedback gates record is evolvable as well, and encoded in the genes describing each feedback gate. Observe that the feedback itself is not supplied externally from a supervisor, but the MB itself must evolve a mechanism to detect which actions have a positive or negative outcome. Based on this, the MB then applies feedback to their feedback gates internally.

Unfortunately, feedback gates and probabilistic logic gates violate a critical requirement of information integration theory 3.0 [2]. To accurately compute Φ, components must be decomposable [22]. Decomposable gates receive inputs and have more than one output. However, the outputs of these gates need to be probabilistically independent from one another as they are not allowed to share information. For this reason, we created probabilistically independent (called *decomposable*) versions of all the gates, including feedback gates (see the Methods for a detailed description of decomposable gates). In addition to facilitating Φ analysis, they provide a small benefit for evolutionary adaptation. For example, in [21] it took 500,000 generations for animats to evolve, but we show with decomposable gates the same performance after 200,000 generations.

A key part of MB evolution is that their interaction with their environment requires them to be embodied [23]. These learning animats evolve to use an internal feedback mechanism to change the function of their logic gates. In this way, we can compare animats that are naïve about their task with those that have experienced the task before. These experiences can structurally change the neural substrate, and consequently Φ may change.

### 1.2. Learning Environment

**Figure 1:**
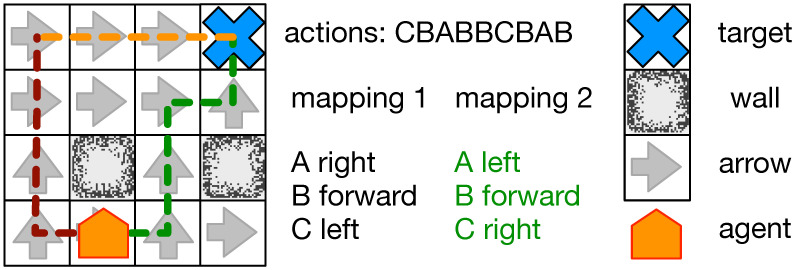
Overview of the navigation task. An animat (orange) must navigate towards a goal (blue cross). Each cell contains an arrow (gray arrow) indicating the shortest path towards the goal, circumventing obstacles and walls (black/gray blocks). However, the animat has no definite control over its body and can only choose actions which are mapped to movements. An example sequence of action, as seen in the figure (CBABBCBAB) might lead towards the goal (mapping 2 green path) while given a different mapping (mapping 1 dark red path) it might lead the animat astray. If the animat can integrate the information it collected while navigating erroneously (dark red path), it could learn the correct mapping and proceed to the goal (orange path).

In previous work, we evolved animats who learn to solve a navigation task (see Figure 1). We now use that framework to investigate the effect of learning on Φ. To evolve these animats, we placed them in a 2D lattice (64 cells wide and 64 cells long) environment with walls as exterior-facing cells and a few of the inner cells. From the remaining cells, we selected a random one as the goal position and computed Dijkstra paths [24] to the goal from all other cells. Each of the cells on the path is assigned a direction indicating the next cell on the path to guide the animat’s navigation. Simply put, the animat only needs to follow this series of directions from cell to cell to reach the goal. The animat was randomly placed 32 steps from the goal and oriented in a random direction. The animat was then allowed to reach the goal as many times as it could within 512 timesteps. At each step, the animat could either move forward, turn 90${}^{\circ }$, or wait. If the animat reached the goal, then the animat was replaced at a random cell 32 steps away from the goal.

This presents a very easy task and would not require animats to remember or learn anything. To improve the complexity of the task and to require animats to remember and learn, the animats were not allowed to directly choose to move or turn but instead had to choose from a set actions associated with results. An animat would choose one of four actions (A, B, C, or D), which were each associated with a different one of four results: turning 90${}^{\circ }$ left, turning 90${}^{\circ }$ right, doing nothing, or moving forward. This indirection of action to result allowed different mappings between those actions and their associated results, preventing the agents from evolving for any one particular mapping. There are 24 possible combinations of mappings from actions to results, so every time an animat was placed in a new variation of the environment the next combination was used. For example, in one mapping the action A may result in a left turn, B a right turn, C nothing, and D a movement forward. In the next map, the selection of A moves the animat forward, B does nothing, C leads to a left turn, and D leads to a right turn. After each animat in the population experienced all 24 combinations, tournament selection was used to determine which animats reproduced. Over 200,000, generations animats evolved the ability to properly navigate their environment given unknown action-result mappings. To do so, they needed to learn by trial and error how actions mapped to movement and rotation.

### 1.3. Different Ways of Measuring Φ

Using animats evolved in this environment, we can now measure the amount of Φ. However, the method used to measure Φ has changed over the history of information integration theory. The latest version of $\Phi^{Max}$ [9] seems to be the most appropriate method to measure the amount of information integration, because this version can measure Φ for a particular moment in time. Unfortunately, computing $\Phi^{Max}$ is computationally costly, and current implementations take about 24 h for one animat at one point in time just to find the main complex (nodes that are essential for the integration of information). Here, we need to measure $\Phi^{Max}$ for hundreds of data points in hundreds of replicates over hundreds of generations, precluding us from using $\Phi^{Max}$.

Fortunately, $\Phi_{atomic}$ (see Equation (1)) is a computationally much simpler measure. While the states of all components at each time point still need to be known, the computation of $\Phi_{atomic}$ does not require an iteration over all parts of all partitions—a calculation with a complexity of $O(B_{n}N^{2})$, where $B_{n}$ is the Bell number of *n* (nodes), which scales super exponentially. Instead, using $\Phi_{atomic}$ reduces the problem to a complexity of $O(n^{2})$ (for a more detailed description of $\Phi_{atomic}$, see [7]).

(1) $$\Phi_{atomic}=SI_{atomic}-I$$

$\Phi_{atomic}$ depends on the definition of information that is processed by the entire system over time. To do this, we use joint random variables $X=X^{\left(1\right)}x^{\left(2\right)}\cdots X^{\left(n\right)}$, where $X^{\left(i\right)}$ represents the system nodes changing the system state over time (*t*). Each $X_{t}$ is defined by the probability $p(x_{t})$ to observe $X_{t}$ in state $x_{t}$. Time changes each node *i* as $X_{0}^{\left(1\right)}\to X_{1}^{\left(i\right)}$ and each $X_{t}^{\left(i\right)}$ is described by probability distribution $p(x_{j}^{i})$.

Therefore, for any time step *t* to t+1, we can calculate the information processed by the system as

(2) $$\begin{array}{c} I\left(X_{t}:X_{t+1}\right)=\sum_{x_{t},x_{t+1}}p\left(x_{t},x_{t+1}\right)\log\frac{p(x_{t},x_{t+1})}{p\left(x_{t}\right)p\left(x_{t+1}\right)}. \end{array}$$

Thus, $\Phi_{atomic}$ is a quantification of the information processed by the system that is more than the sum of the information processed by the individual units:

(3) $$\begin{array}{c} \Phi_{\mathrm{atomic}}=I\left(X_{t}:X_{t+1}\right)-\sum_{i=0}^{n}I\left(X_{t}^{\left(i\right)}:X_{t+1}^{\left(i\right)}\right)+I. \end{array}$$

where $I(x_{t}^{\left(i\right)}:X_{t+1}^{\left(i\right)})$ represents the information processed by node *i*, and I is the nonindependence between the network variables [25,26,27,28,29]:

(4) $$\begin{array}{c} I=\sum_{i=0}^{n}H\left(X_{t}^{\left(i\right)}\right)-H\left(X_{t}\right). \end{array}$$

With this “more than the sum of its parts” system information, and spatial integration of nonindependence, $\Phi_{atomic}$ captures both temporal and spatial network information characteristics. These terms together simplify to

(5) $$\begin{array}{c} \Phi_{\mathrm{atomic}}=\sum_{i=0}^{n}H\left(X_{t}^{\left(i\right)}|X_{t+1}^{\left(i\right)}\right)-H\left(X_{t}|X_{t+1}\right) \end{array}$$

It has also been shown that $\Phi_{atomic}$ is a very good correlate with more complex versions of Φ [7,8], making it an ideal substitute, with the exception of no previous method to use on individual time points. However, here we discovered a way to calculate $\Phi_{atomic}$ for individual time points. In previous experiments [7,8], $\Phi_{atomic}$ was calculated by recording the animat’s brain states (*X*) at every time point (*t*), and one needs all of these $X_{t}$ to $X_{t+1}$ transitions in order to compute $\Phi_{atomic}$. However, for each time point *t* we can compute all possible state-to-state transitions the animat’s brain could theoretically experience. This is done by computing the state-to-state transition probability matrix (TPM) at time point *t* assuming $H_{t}$ to be a uniform random variable encompassing all possible brain states, and $H_{t+1}$ to be defined by the TPM. Here, we derive the TPM for each time point by stochastic sampling. Observe that a machine or brain that would not change during its lifetime will have the same TPM for each time point. Only if the probabilities defining the state-to-state transitions change, for example as it happens here due to learning, a machine would be able to have different TPMs for each time point. We define the $\Phi_{atomic}$ measurement performed in this manner as $\Phi_{maxH}$. In this way, we can quantify an animat’s ability to integrate information before, during, and after learning has affected change.

## 2. Results

**Figure 2:**
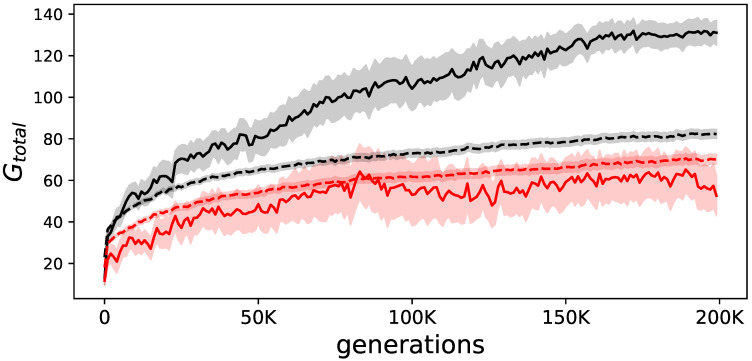
Evolution of goal-reaching. The solid lines show the average performance of the elite animats, while the dashed lines show the average performance of all animats. In black, the summation of goals reached ($G_{total}$) over all 24 environmental variations, in red the same but when measured in animats that were prevented from using their evolved internal feedback mechanism. The difference between the solid (elite) and dashed black line (all animats) illustrates how much better elite animats perform. The shadows indicate the standard error of the means.

Animats were evolved to perform a navigation task for which they had to use environmental clues to determine the correct motor outputs, and thus the correct behavior. Given the difficulty of the task, only 13 of all the 200 replicate experiments resulted in animats that performed well at the end of evolution. Specifically, all animats were tested on all possible 24 variations of the environment, one with learning (allowing them to take advantage of their evolved internal feedback mechanisms), and one without learning. After 200,000 generations, only those animats that reached the goal two-fold more with learning than without were considered “elite.” From these 13 elite performers, all animats on the line of decent (LoD, see Methods for details) were evaluated in the same environment to analyze Φ. As expected, animats evolved the ability to reach the goal, and used their internal feedback mechanism to do so. The difference between performance with and without internal feedback between the elite animats and all animats shows this (see Figure 2). We further find that elite animats perform on average worse when their internal feedback mechanism is disabled when compared to all other animats. This could be due to the different behavioral strategies. Elite animats obviously have evolved the ability to learn from their environment, and are thus probably more dependent on their feedback mechanisms as opposed to the not so well evolved other animats.

**Figure 3:**
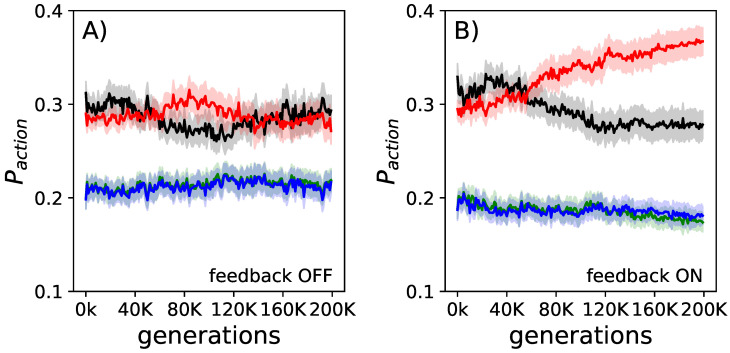
Evolution of goal-oriented action selection. The fraction of actions used (black idle, red forward, green left turns, blue right turns) over the course of evolution averaged over all elite performers. In panel (**A**), measured when the internal feedback mechanism was turned off, we find almost no change in action selection preference. In panel (**B**), measured when the internal feedback mechanism was turned on, we find that the number of turns and idles are reduced, while the number of forward movements becomes increased. The shadows represent the standard error of the means for each action.

Over the course of evolution, animats optimized their ability to navigate the environment, and to learn how the possible choices map to their movements. As expected, animats learn to move more deliberately through the environment, by reducing the number of unnecessary turns, and idle behavior, while moving forward more often (see Figure 3). These changes in action frequencies are slightly different to those observed before [21] for agents that used non-decomposable feedback gates. We assume that these differences are due to the small functional differences necessary to ensure decomposability. However, they are neglectable.

**Figure 4:**
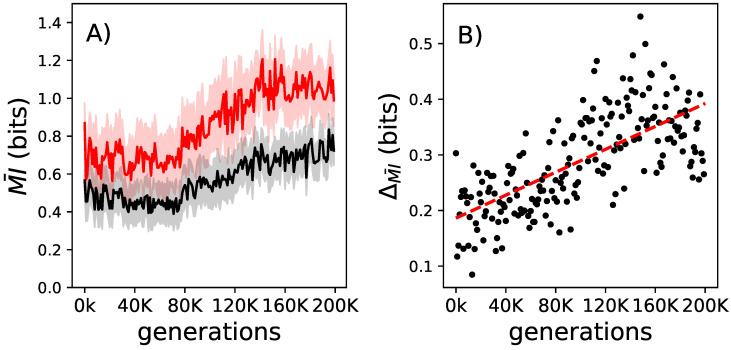
The change to mutual information conveyed by decomposable logic gates over their lifetime and over evolutionary time. Panel (**A**): In black is the average mutual information that the decomposable logic gates convey for all elite performers at the beginning of their lives. In red is the same but measured at the end of the animats’ lives. The shadows indicate the standard error of the means for those measurements. Panel (**B**): The lifetime increase of the mutual information that decomposable logic gates convey between birth and the end of life ($\Delta_{\bar{MI}}={\bar{MI}}_{death}-{\bar{MI}}_{birth}$ measured for all elite performers on the line of decent. The red dashed line shows the linear regression trend ($r^{2}=0.697$ and a *p*-value of 2.034 × 10^−30^).

Decomposable feedback gates must change their probability matrix to allow for lifetime learning. The mutual information that these gates convey between their inputs and outputs should increase over the lifetime, and we expect that the delta of this change also increases over evolutionary time, as observed before [21] for non-decomposable logic gates. As expected, the mutual information is increasing over the lifetime of an animate and due to evolution (see Figure 4).

To obtain an additional impression of how animats behave and how their ability to learn changes over evolutionary times and over their lifetime, see the Supplementary Movie.

### 2.1. Effects of Evolution on Φ

**Figure 5:**
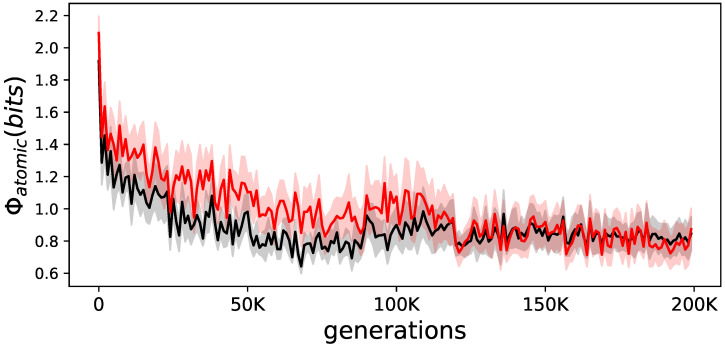
Evolution of $\Phi_{atomic}$ for elite animats. The average $\Phi_{atomic}$ for elite animats on the LoD. In black are the animats that were allowed to learn; in red are the same animats but prevented from learning during their lifetime. The shadows indicate the standard error or the means.

Earlier experiments with $\Phi_{atomic}$ showed increases over evolutionary time [7,8,9,11]. Here, it seems that on average, $\Phi_{atomic}$ becomes lower, and animats that are not allowed to learn have a slightly higher value (see Figure 5).

**Figure 6:**
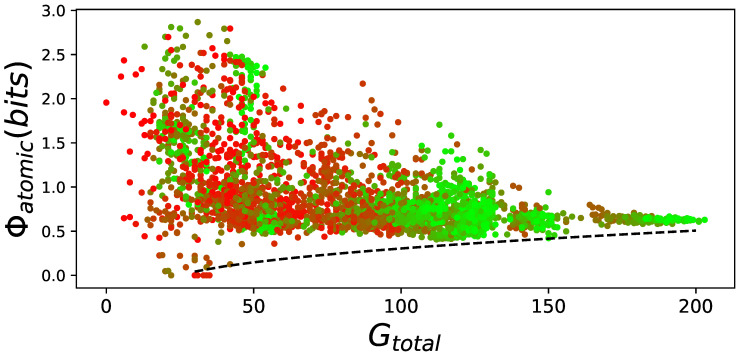
$\Phi_{atomic}$ versus performance on the LoD. For all elite performers on the LoD, $\Phi_{atomic}$ was plotted against their performance (total number of goals reached, pooled over all possible 24 action mappings $G_{total}$). The LoD generation time point is indicated by a color gradient from red (beginning of evolution) to green (end of adaptation). The lower bound relation of $\Phi_{atomic}$ to performance $G_{total}$ is indicated by a dashed black line (hand drawn, since there is no proper way to fit a lower bound). This figure visualizes how $\Phi_{atomic}$ changes due to evolution for all elite performers. The better animats become, the more goals they reach, and the further right their data points move on the X-axis. Similarly, the less $\Phi_{atomic}$ they have, the lower they move on the Y-axis. To incorporate the generations (which is roughly similar to the animats’ performance), each data point is color coded, starting with red at the beginning of evolution, and becoming green at the end of evolution. This behavior is very similar to that found before [7,8].

However, this result can be explained. Random animats can have high values of $\Phi_{atomic}$ regardless of their ability to perform. Only well-performing animats require a minimal amount of $\Phi_{atomic}$. This has been shown before [7,8] and we observe the same phenomena here (see Figure 6). We find the same lower bound, where animats require a minimal amount of $\Phi_{atomic}$ to perform. In addition, we observe that at the end of evolution with learning, variance of $\Phi_{atomic}$ over all animats converges towards zero, whereas without learning evolved animats greatly varied in their ability to integrate information.

### 2.2. Effects of Learning on Φ

**Figure 7:**
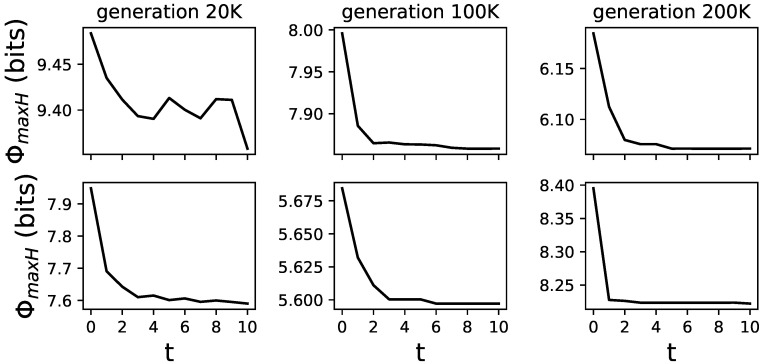
Examples of different animats and how $\Phi_{maxH}$ changes over their lifetime. Six randomly chosen animats and their lifetime changes in $\Phi_{maxH}$, averaged over all 24 actions-effect mappings. Animats were chosen from the LoD at generations 20,000, 100,000, and 200,000. These are representative samples, to illustrate how $\Phi_{maxH}$ changes. All examples can be found in Figure 8.

The addition of feedback gates to MBs allows these brains to change from generation to generation and during their lifetime. This allows us to test how these lifetime changes (learning) affect Φ. We use $\Phi_{maxH}$ to assess an animat’s ability to integrate information during a specific time point in its life. This is possible because $\Phi_{maxH}$ only requires a TPM which can be derived at any time point and does not need an observation of the animat in its environment, which might alter its experience through learning. Animats were given 512 time points in the environment, and we calculated $\Phi_{maxH}$ every 50 time points. As an observation, we find animats to reduce the amount of $\Phi_{maxH}$ over their lifetime (see Figure 7 for examples of differently evolved animats and how $\Phi_{maxH}$ changes over their lifetime).

**Figure 8:**
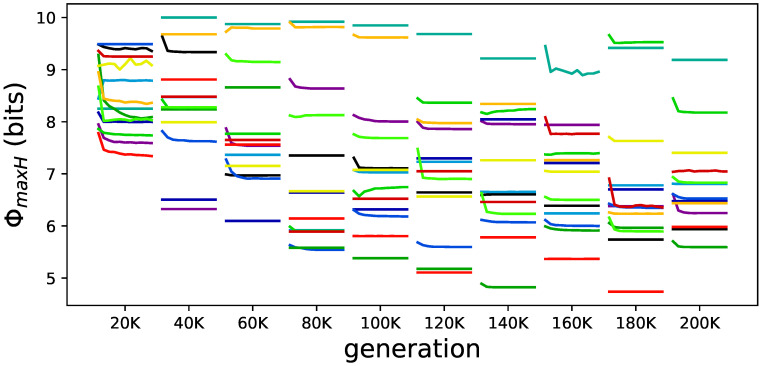
Samples of all elite animats and how $\Phi_{maxH}$ changes over their lifetime for various stages of evolution. Each line represents the change in $\Phi_{maxH}$ experienced by each animat at different times over their evolutionary history (generations are indicated on the X-axis). Each color represents an independent experiment; the Y-axis is kept to scale so that the general reduction of $\Phi_{maxH}$ can be observed.

Beyond this apparent reduction of $\Phi_{maxH}$ over the animats’ lifetime, we also see two additional trends. The first is a general reduction of $\Phi_{max}$ over evolutionary time, similar to the reduction of $\Phi_{atomic}$. The second observation is an additional but total smaller reduction of $\Phi_{maxH}$ over the lifetime of elite animats (see Figure 8).

**Figure 9:**
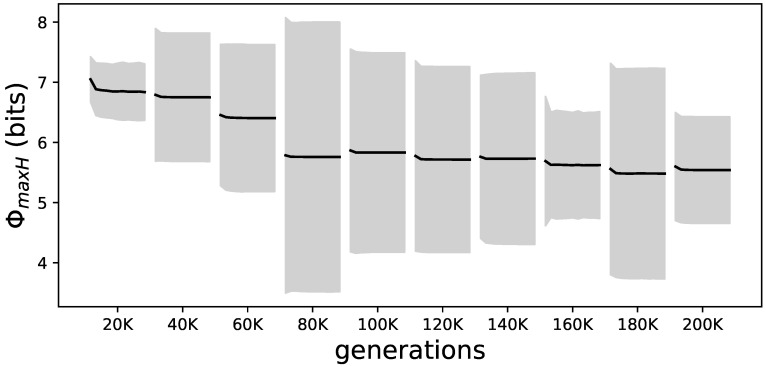
Evolutionary and lifetime changes of $\Phi_{maxH}$ for all elite performers. Samples of the elite performers from the LoD every 20 K generations. Each interval shows the average $\Phi_{maxH}$ from all elite performers over their lifetime as a black line; the gray shadow marks the standard deviation. This figure presents the averages of the values shown in Figure 8 per time point on the LoD. The reduction of $\Phi_{maxH}$ over evolutionary time as well as within each lifetime can be seen.

Specifically, we find that the amount of $\Phi_{maxH}$ at the beginning and end of animats’ lifetimes decreases for all elite performers during evolution (see Figure 9).

**Figure 10:**
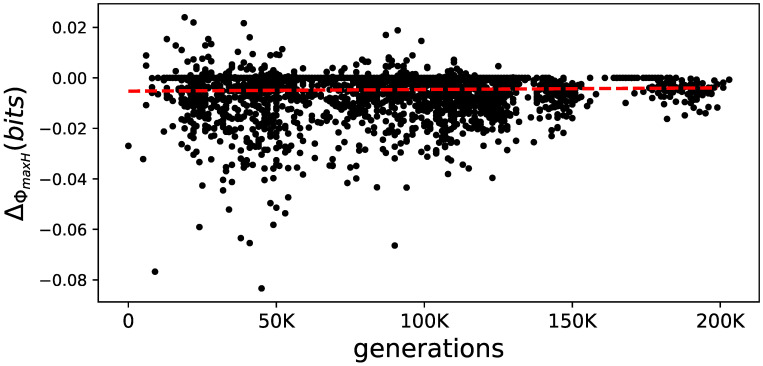
The lifetime change in $\Phi_{maxH}$ over generations. From all elite performers, the change in $\Phi_{maxH}$, measured as the difference in $\Phi_{maxh}$ at the end of their lifetime and the beginning ($\Delta_{G_{total}}$) over generations. The correlation coefficient of 0.224 is indicated as a linear fit as a red dashed line.

In addition, we characterize the reduction of $\Phi_{maxH}$ by fitting a linear function and using the slope of that fit as a proxy for the reduction. This slope correlates with evolutionary time (generations) over all elite performers (see Figure 10). Both types of analysis (Figure 9 and Figure 10) show that the amount of information integration due to learning is reduced and that the reduction itself becomes smaller.

## 3. Discussion

In this work, we measured how lifetime learning influences an animat’s ability to integrate information. We had no intuition of how information integration would respond to learning: increase, decrease, or remain. Because such measurement is currently prohibitively difficult with natural organisms—let alone human beings—we use evolved animats as the next best substitute. Markov Brains controlled these animats, because they were a proven study system used for similar work and thus allow for direct comparisons. The biggest constraint on this type of assessment for any continuous or probabilistic computational system is the necessity to be computationally decomposable. To date, only decomposable probabilistic logic gates [22] have had this feature, and here we introduced decomposable feedback gates. We know of no other system that has this property, is evolvable, and has sufficient neural plasticity to adapt during its lifetime. This precludes other systems for identical studies until their decomposability has been shown (artificial neural networks [30], for example). Regardless, in future work this analysis should be repeated with other computational systems, and eventually on natural organisms. Until then, we conjecture that these results can be generalized to those systems.

Instead of measuring $\Phi^{Max}$ from IIT 3.0 or EI from IIT 2.0, here we used the more computationally tractable $\Phi_{atomic}$ to quantify an animat’s ability to integrate information during its lifetime, and $\Phi_{maxH}$ to measure changes in an animat’s ability to integrate information. We assume that these results should generalize to $\Phi^{Max}$ and hope to use that measure or any successor in the future when computational simplifications can be found.

## 4. Methods

To study how information integration changes over an organism’s lifetime, we created a computational model in which animats are required to navigate in a changing environment. Animats needed to learn how to properly move within this environment. The brain of each animate was a probabilistic finite state machine that consisted of sensor nodes (input), memory nodes (hidden), and action nodes (output). This was implemented by a Markov Brain (MB) [7,12]. The animates were evolved using a genetic algorithm (GA) that selected for individuals that successfully decoded a variety of symbols.

### 4.1. Environment

Populations of 100 animats were evolved in 200 replicates to perform a spatial navigation task [21] described above. Populations could evolve for 200,000 generations testing their performance on all 24 possible variations of that environment at every generation. Upon evolutionary completion, the LoD for every replicate was constructed. These animats were then again tested in the same environments but their internal feedback mechanism was disabled. The difference in performance between using feedback and not being allowed to use feedback was used as an indicator to identify elite performers. Only animats that on average reached each of the navigation goals two times more often with feedback than without were considered elite. This resulted in 13 independent evolutionary experiments.

### 4.2. Animat

Animats’ brains consisted of 16 states: 4 input sensors, 2 output motors, and 10 hidden states. Each input sensor was assigned a direction to detect: sensor 0 fired when the animat was told to stay put, 1 indicated turn right, 2 go forward, and 3 turn left. As described above, the environment was composed of tiles with arrows that point towards the shortest path to the goal. Sensors could read the arrow of the tile that the animat was currently on. When the sensor detects its given direction, the state was set to 1, otherwise the state was 0. For example, if the map indicated the animat’s next closest step was to the left, its first four states would then be: 0,0,0,1.

### 4.3. Line of Descent

The LoD for an experiment was determined by choosing a random animat in the final generation and tracing its lineage to the first generation [31]. Through this method, the most recent common ancestor (MRCA) and all mutations that reached fixation in the population were recorded.

### 4.4. Feedback Gates

The key feature of feedback gates is that every input *i*, which results in output *o* is defined by a probability that has the potential to change with each update due to the feedback received. Each time these numbers change, so does the amount of predictive information shared between nodes, resulting in a change to $\Phi_{atomic}$ [7]. It is important to note that the typology, or the connections, between nodes does not change during the lifetime of an animat; only the information contained in the connection changes. Topological changes, or changes to the start condition of the probabilities, however, can occur due to mutations from one generation to the other.

### 4.5. Decomposable Feedback Gates

Decomposable feedback gates superset includes features from feedback gates, decomposable gates, and probabilistic gates. A probabilistic gate relies on a probabilistic mapping between input and output patterns. However, each input pattern is associated with a separate set of probabilities over all output patterns. Decomposable gates are based on the design of probabilistic gates. To ensure decomposability of outputs for a single input pattern, the output patterns must have probabilities such that the probabilities of single outputs are independent. In other words, if an input pattern could produce output 0 0 with probability 0.36, 0 1 with 0.16, 1 0 with 0.16, and 1 1 with 0.04, it is possible to compute the independent probabilities for each of the two bits *a* and *b* of 0.4 and 0.4. 0 0 (both bits off) = (1−a)*(1−b), 0 1 = (1−a)*b, 1 0 = a*(1−b), and 1 1 = a*b. However, if the output pattern probabilities related to a single input pattern were 0.02, 0.48, 0.48, and 0.02, then it would be impossible to determine the independent probabilities for each bit because their likelihoods are dependent on the state of one another. This decomposability of independence is important when we use the information theoretic analysis of Φ so as not to invalidate the fundamental assumptions of independence.

The probabilistic gates for an animat are constructed upon the *birth* (construction) of the organism, and derive their probabilities directly from the genome. For a decomposable gate, the genome provides the *factors* for each input pattern that are then multiplied together to produce a full row of output pattern probabilities. In this way, each input pattern will probabilistically produce any of the possible output patterns such that the probability of individual output-bit activations within each pattern is independent across all patterns associated with that input.

To allow learning via feedback over a lifetime, we add to the design of each gate a positive feedback connection to one of the states (input, hidden, output), and a negative feedback connection to one of the states. When the input bit for positive feedback is activated, the probability for the previous input–output activation is increased by a small amount, and one of the other output pattern probabilities randomly chosen for that input is decreased by the same delta. Negative feedback happens in a similar way when the input bit wired for negative feedback is activated. This configuration allows feedback to time *t* of the time point t−1. Additionally, we allow feedback on further time points into the past t−2, t−3, and so on, each with their own feedback deltas. Evolution controls how any of these feedback mechanisms are used. For every gate, the genome encodes the following: which two states are used for positive and negative feedback, the feedback history length between 0 and 3 time points, and the positive and negative feedback deltas between 0 and max probability. Since each individual probability is modified separately due to feedback, and then used to reconstruct the probability table of the gate decomposable feedback, gates remain decomposable even after feedback is applied.

## 5. Conclusions

From earlier experiments, we understood how Φ changes over evolutionary time: there is a lower bound such that performance only increases together with Φ, and that increasingly complex environments also require an increase in Φ. While we knew that learning increased the animats’ performance, we did not know how lifetime learning would affect Φ. Furthermore, it was unclear if the increase in performance was due to streamlining the computation, thus reducing integration, or if better performance required more integration of information? To determine how the integration of information was affected by learning, we tested how $\Phi_{atomic}$ behaves over evolutionary times as well as how it is affected by lifetime learning. The tests performed here confirm all prior observations about Φ.

Over the course of the animat’s life, $\Phi_{maxH}$ is reduced, suggesting that at birth the animat had to integrate more information to streamline the computation. As a result, after learning, the animat integrates less information. Applying this idea to the soccer player analogy implies that the well-trained player integrates less information than she did at the beginning of her career.

The second interesting observation is that the reduction of information integration becomes further minimized over evolutionary time. The more evolved an animat becomes, the less it must reduce its information integration due to learning. This suggests that the learning process itself becomes optimized to reduce the change to information integration. This seems surprising. Because learning reduces $\Phi_{maxH}$, one might assume that better learning correlates with more reduction. However, our results contradict this notion and instead show that the better an animat learns, the less it reduces $\Phi_{maxH}$. This phenomenon might result from faster learning being more advantageous than slower learning in this particular environment. The larger the probability table change necessary, the slower learning is. Consequently, there is an evolutionary advantage to change the system as little as possible, which might explain why animats adapt $\Phi_{maxH}$ minimally at the end of evolution. This phenomenon could also explain the observation that $\Phi_{atomic}$ converges to the smallest possible value (see Figure 5) instead of being more distributed towards the end of evolution.

**Figure 11:**
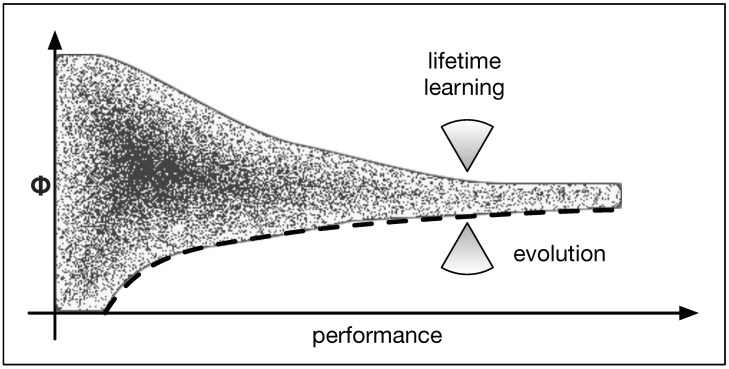
Illustration of two forces. Over the course of evolution, we find Φ to necessarily increase to allow for higher levels of performance. This is illustrated by the lower bound to Φ (dashed line) and suggests that evolution seeks to increase Φ (lower of the two arrows). At the same time, lifetime adaptations are time-consuming in this environment and should thus be minimized. Consequently, we hypothesize that the second influence due to learning reduces Φ (upper arrow).

Therefore, we hypothesize that the behavior of Φ depends on two “forces” (influences). Evolution optimizes the function of the animat which is only limited by its ability to integrate information. Consequently, evolution increases Φ over time (see Figure 11 lower arrow). On the other hand, lifetime learning streamlines the computation and reduces the amount of integration. The degree to which this needs to be done is further minimized to improve performance and to accelerate learning. This creates a second force which minimizes Φ (see Figure 11 upper arrow). These two influences now force Φ to converge.

It is entirely possible that the phenomenon we observe is dependent on the specific task, type of evolutionary optimization, and the life time learning mechanism implemented here. Other forms of learning for other tasks, particularly those where time is not of the essence, may behave differently. Investigating the changes to Φ due to learning (or other forms, such as back propagation or reinforcement [30]) presents an interesting opportunity for future research.

## Supplementary Materials

The following are available at https://www.mdpi.com/1099-4300/21/5/524/s1.
